# Solution structure of the major G-quadruplex formed in the human VEGF promoter in K^+^: insights into loop interactions of the parallel G-quadruplexes

**DOI:** 10.1093/nar/gkt784

**Published:** 2013-09-04

**Authors:** Prashansa Agrawal, Emmanuel Hatzakis, Kexiao Guo, Megan Carver, Danzhou Yang

**Affiliations:** ^1^Department of Pharmacology and Toxiocology, College of Pharmacy, University of Arizona, 1703 E. Mabel St, Tucson, AZ 85721, USA, ^2^Department of Chemistry, University of Arizona, Tucson, AZ 85721, USA, ^3^BIO5 Institute, University of Arizona, Tucson, AZ 85721, USA and ^4^The Arizona Cancer Center, Tucson, AZ 85724, USA

## Abstract

Vascular endothelial growth factor (VEGF) proximal promoter region contains a poly G/C-rich element that is essential for basal and inducible VEGF expression. The guanine-rich strand on this tract has been shown to form the DNA G-quadruplex structure, whose stabilization by small molecules can suppress VEGF expression. We report here the nuclear magnetic resonance structure of the major intramolecular G-quadruplex formed in this region in K^+^ solution using the 22mer VEGF promoter sequence with G-to-T mutations of two loop residues. Our results have unambiguously demonstrated that the major G-quadruplex formed in the VEGF promoter in K^+^ solution is a parallel-stranded structure with a 1:4:1 loop-size arrangement. A unique capping structure was shown to form in this 1:4:1 G-quadruplex. Parallel-stranded G-quadruplexes are commonly found in the human promoter sequences. The nuclear magnetic resonance structure of the major VEGF G-quadruplex shows that the 4-nt middle loop plays a central role for the specific capping structures and in stabilizing the most favored folding pattern. It is thus suggested that each parallel G-quadruplex likely adopts unique capping and loop structures by the specific middle loops and flanking segments, which together determine the overall structure and specific recognition sites of small molecules or proteins.

LAY SUMMARY: The human VEGF is a key regulator of angiogenesis and plays an important role in tumor survival, growth and metastasis. VEGF overexpression is frequently found in a wide range of human tumors; the VEGF pathway has become an attractive target for cancer therapeutics. DNA G-quadruplexes have been shown to form in the proximal promoter region of VEGF and are amenable to small molecule drug targeting for VEGF suppression. The detailed molecular structure of the major VEGF promoter G-quadruplex reported here will provide an important basis for structure-based rational development of small molecule drugs targeting the VEGF G-quadruplex for gene suppression.

## INTRODUCTION

The human vascular endothelial growth factor (VEGF) is a pluripotent cytokine and a key regulator of angiogenesis. VEGF plays an important role in tumor survival, growth and metastasis (1,2). It binds to VEGF receptors on the surfaces of endothelial cells to promote the formation of new blood vessels, or angiogenesis, which can promote tumor growth by providing oxygen and nutrients as well as provide escape routes for disseminating tumor cells (3,4). VEGF overexpression is frequently found in a wide range of human tumors (5–8) and can be induced by the loss or inactivation of tumor suppressor genes (9), the activation of oncogenes (10), external stimuli such as hypoxia and cytokines (11,12) and transcriptional upregulation, which is controlled by the *cis*-acting elements and transcription factors (5–9,13). Anti-VEGF therapy has been actively pursued for cancer therapeutics in a variety of forms, including antibodies, ribozymes, immunotoxins and small molecule inhibitors (14–23).

The G-quadruplexes formed in oncogene promoters have been shown to be potential targets for small molecule drugs (24–26). Most recently, the existence of DNA G-quadruplex has been visualized on chromosomes in human cells using a G-quadruplex-specific antibody (27). One region proximal to the transcription initiation site, a 39-bp polyG/polyC region located −88 to −50 bp relative to the transcription initiation site, has been shown to be functionally significant in VEGF transcriptional activity with multiple transcription factor binding sites, including three potential Sp1 binding sites (13). This region has been shown to be highly dynamic in conformation and can form DNA G-quadruplex secondary structure on the G-rich strand, as demonstrated by *in vitro* and plasmid footprinting with dimethyl sulfate (DMS), DNase I and S1 nuclease in K^+^ (28,29), and by *in vivo* DMS footprinting using A498 kidney cancer cells that overexpress VEGF (30). The formation of DNA G-quadruplex structure is clearly enhanced by G-quadruplex-interactive agents (28), which can repress VEGF expression in human tumor cells (31), suggesting that the VEGF G-quadruplex is amenable to small molecule drug targeting for VEGF suppression. A detailed molecular structure of the major VEGF promoter G-quadruplex will be important for structure-based rational development of small molecule drugs (32).

We report here the nuclear magnetic resonance (NMR) structure of the major G-quadruplex formed in the human VEGF promoter in K^+^ solution. Our NMR study unequivocally demonstrated that the major intramolecular G-quadruplex formed in the VEGF promoter in K^+^ is a parallel-stranded structure with 1:4:1 loop-size arrangement. We have found that the middle 4-nt loop interacts with the 5′ flanking residues to form a specific capping structure, a salient feature as this interaction is specific to the VEGF sequence and differs from those other parallel-stranded structures. Together with the 5′-flanking segment, the 4-nt middle loop appears to play a central role in forming the specific capping structure that likely determines this most favored folding pattern. Parallel-stranded G-quadruplexes have been found to be common in the human promoter sequences. Significantly, our results indicate that each parallel structure is likely to adopt unique capping and loop structures by the specific flanking sequences and middle loops, which together determine the stability of the overall G-quadruplex structure and potential specific interactions with small molecules or proteins.

## MATERIALS AND METHODS

The synthesis and purification of DNA oligonucleotides was done as described earlier (33–37). Water samples were prepared in 90%/10% H_2_O/D_2_O solution. Samples in D_2_O were prepared by repeated lyophilization and final dissolution in 99.96% D_2_O. The final NMR samples contained 0.1–2.5 mM DNA in 25 mM K-phosphate buffer (pH 7.0), 70 mM KCl.

Circular dichroism (CD) spectroscopic study of the oligonucleotides was performed on a Jasco J-810 spectropolarimeter (Jasco Inc., Easton, MD, USA) equipped with a thermoelectrically controlled cell holder as described previously (38). The quartz cell of 1 mm optical path length was used. A blank sample containing only buffer was used for the baseline correction. CD spectroscopic measurements were the averages of three scans collected between 200 and 350 nm. The scanning speed of the CD instrument was 100 nm/min, and the response time was 1 s. T_m_ values were measured by CD melting and annealing experiments performed at 265 nm for three repeats, with a heating or cooling rate of 2°C/min, respectively.

NMR experiments were performed on a Bruker DRX-600 MHz spectrometer as discussed earlier (33–37). Stoichiometric titration of the unfolded and folded strands as a function of total strand concentration from 0.01 to 0.1 mM was performed at 75°C (melting point) (39). The guanine H1 imino protons, one-bond coupled to N1, and H8 protons, two- bond coupled to N7, can be unambiguously assigned by 1D ^15^N-edited heteronuclear multiple quantum coherence (HMQC) experiments (40). For this purpose, site-specific labeled DNA synthesis with 6% ^15^N-labeled-guanine phosphoramidite (41) was used. The 1D GE-JRSE HMQC experiments were used for measuring ^15^N-edited spectra (40) to identify guanine imino and H8 protons. The 1D variable temperature (VT) proton NMR experiments were done in the range from 1°C to 80°C. Homonuclear 2D-NMR experiments double quantum filtered-correlation spectroscopy (DQF-COSY), total correlation spectroscopy (TOCSY) and nuclear overhauser effect spectroscopy (NOESY) were collected at 5, 15 and 25°C for complete proton resonance assignment in water and D_2_O solution. The contribution from J-modulation and zero quantum coherence effect was suppressed by using z-gradient filter having gradient strength 20% and a duration of 1 ms. The NMR experiment for samples in water were performed using Jump-return spin-echo water suppression technique in which water peak was suppressed with maximum intensity tuned to 11 ppm (42). Relaxation delays were set to 2.5 s. The acquisition data points were set to 4096 × 512 (complex points). Peak assignments and integrations were achieved using the software Sparky (UCSF). Non-exchangeable protons were estimated based on the Nuclear Overhauser Effect (NOE) cross-peak volumes at 50–300 ms mixing times, with the upper and lower boundaries assigned to ±20% of the estimated distances. Distance restraints for the unresolved cross-peaks were set with looser boundaries of ±30%. The cytosine base proton H6-H5 distance (2.45 Å) was used as a reference distance. The distances involving the unresolved protons, e.g., methyl protons, were assigned using pseudo-atom notation to make use of the pseudoatom correction automatically computed by X-PLOR.

The structure of Pu22-1213T was calculated using X-PLOR (43). Metric matrix distance geometry and simulated annealing calculations were carried out in X-PLOR (43) to embed and optimize 100 initial structures based on an arbitrary extended conformation of the single-stranded Pu22-1213T sequence to produce a family of 100 DG structures, as described previously (33,34). The experimentally obtained distance restraints and G-tetrad hydrogen bonding distance restraints were included during the calculations. All of the 100 molecules obtained from the distance geometry simulated annealing (DGSA) calculations were subjected to NOE-restrained Simulated Annealing refinement in X-PLOR (43) with a distance-dependent dielectric constant. A total of 407 NOE distance restraints were introduced into the NOE-restrained structure calculation with a force constant of 20 kcal mol^−^^1 ^Å^−^^2^. Hydrogen bond restraints were applied to the G-tetrads, using a quadratic energy function with a force constant of 100 kcal mol^−^^1 ^Å^−^^2^. A low-level planarity restraint (2 kcal mol^−^^1 ^Å^−^^2^) was also applied on the G-tetrad in the simulated annealing step of the structure calculation. The planarity restraints were removed in the final molecular dynamics simulation with energy minimization. Dihedral angle restraints were used to restrict the glycosidic torsion angle for the experimentally assigned anti conformation bases and for tetrad-guanines. The 30 best molecules were selected based both on their minimal energy terms and number of NOE violations and were further subjected to NOE-restrained molecular dynamics calculations at 300 K for 25 ps. The coordinates saved at every 0.1 ps during the last 2.0 ps of NOE-restrained molecular dynamics calculations were averaged, and the resulting averaged structure was subjected to further minimization until the energy gradient of 0.1 kcal mol^−^^1^ was achieved. The 10 best molecules were selected based both on their minimal energy terms and number of NOE violations with the mean rms deviation of 1.10 Å for the family of 10 ensemble structures. For the G-quadruplex formed in the wild-type VEGF_Pu22 sequence, we took the G-quadruplex formed in Pu22-T12T13 as the starting structure and replaced T12 and T13 with the wild-type G12 and G13 residues. This structure was then subjected to energy minimization followed by unrestrained molecular dynamics simulation for 25 ps at 300 K.

## RESULTS

### The major G-quadruplex formed in VEGF Promoter in K^+^ solution adopts a parallel-stranded structure with 1:4:1 loop-size arrangement

**Figure 1.**
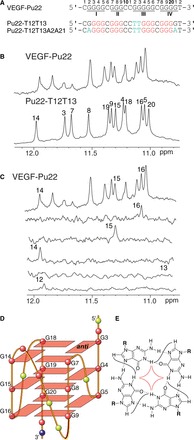
(**A**) The promoter sequence of VEGF and its modifications. VEGF-Pu22 is the 22mer wild-type G-rich sequence needed for quadruplex formation; the four G-runs are numbered. Pu22-T12T13 and Pu22-T12T13A2A21 are modified Pu22 sequences with mutations shown in cyan. Pu22-T12T13 and Pu22-T12T13A2A21 adopt the major 1:4:1 parallel-stranded structure investigated in this study. The numbering system is shown above VEGF-Pu22. (**B**) The imino region of 1D ^1^H NMR spectra of the wild-type VEGF-Pu22 and Pu22-T12T13. (**C**) The imino region of 1D ^1^H NMR spectra of the wild-type VEGF-Pu22. Imino proton assignments of G12-G16 using 1D ^15^N-edited HMQC on site-specific-labeled VEGF-Pu22 at each of G12-G16 are also shown. Conditions: 25 mM K-phosphate, 70 mM KCl (pH 7.0), 25°C. (**D**) Schematic drawing of the major 1:4:1 G-quadruplex formed in VEGF-Pu22 (G = red, C = yellow, T = blue). (**E**) A G-tetrad with H1-H1 and H1-H8 connectivity pattern detectable in NOESY experiments.

The G-rich strand of this VEGF proximal promoter region contains five guanine-runs. Using electrophoresis mobility shift assay (EMSA), DMS footprinting and DNA polymerase stop assay in K^+^ solution, it has been shown that the G-quadruplex formed in this region involves only the 5′ four successive G-runs (VEGF-Pu22, Figure 1A) (29,31), which contain four (G2-G5), three (G7-G9), five (G12-G16) and four (G18-G21) guanines, respectively. VEGF-Pu22 can form multiple loop isomers. The wild-type VEGF-Pu22 forms a clear predominant G-quadruplex structure in 95 mM K^+^ solution, as shown by a set of imino proton peaks at 10.5–12 ppm in ^1^H NMR, characteristic of G-tetrad guanines (Figure 1B). The CD spectrum of VEGF-Pu22 showed a positive peak ∼265 nm and a negative peak at 240 nm (Supplementary Figure S1), characteristic of a parallel-stranded G-quadruplex structure (38). We prepared the wild-type VEGF-Pu22 sequence with 6% site-specific incorporation of ^15^N-labeled-guanine at each guanine of the 5 G-run G12-G16 (Figure 1A). The imino protons of G14, G15 and G16 were clearly detected in 1D ^15^N-edited HMQC experiments, whereas the imino proton of G12 was weak and the imino proton of G13 was missing (Figure 1C); the imino proton of G13 was not detected even at 2°C (Supplementary Figure S2), indicating that the major conformation formed in the wild-type VEGF-Pu22 does not involve G12 and G13 in the G-tetrad formation. Thus, the folding topology of the major G-quadruplex formed in VEGF-Pu22 is a parallel G-quadruplex with a 1:4:1 loop-size arrangement (Figure 1D). This major VEGF G-quadruplex can be isolated by the sequence Pu22-T12T13, with G-to-T mutations at positions 12 and 13 (Figure 1A). Pu22-T12T13 gave rise to a well-resolved ^1^H NMR spectrum in 95 mM K^+^ solution (Figure 1B) and was used for NMR structure determination.

To determine the effect of loop and flanking residues, we have tested various modified VEGF sequences by ^1^H NMR (Figures 1B and Supplementary Figure S3). The spectrum of Pu22-T12 with G12-to-T mutation is almost the same as that of the wild-type VEGF-Pu22, indicating that G12 is involved in neither the tetrad formation nor the capping structure. The spectrum of Pu22-T12T13 is similar to that of the wild-type VEGF-Pu22, with the G7 imino proton down-field shifted, likely due to a smaller ring-current effect of T13 than that of G13 in the capping structure (see later in the text). The spectrum of Pu22-T12T13A2 showed a shifted G18 imino proton, likely caused by a different base pair conformation (T13:A2) of this modified sequence, whereas Pu22-T12T13A2A21 showed additionally shifted G20 and G16 imino protons, likely due to the mutated A21 base.

### The less stable 1:2:3 loop isomer can also be isolated in a modified VEGF sequence in K^+^ solution

Our result is consistent with the previous DMS footprinting data, which show that the 1:4:1 loop isomer is the predominant G-quadruplex formed in the wild-type VEGF promoter sequence in K^+^ solution (29). It was suggested by DMS footprinting that a minor conformation, the 1:2:3 G-quadruplex (Supplementary Figure S4A), could also be formed (29). The 1:2:3 G-quadruplex needs G12 and G13 in the core G-tetrads and can be isolated using the Pu22-T15T16 sequence (Supplementary Figure S4A). Although the ^15^N-edited HMQC experiments of the wild-type sequence VEGF-Pu22 in K^+^ did not detect the formation of the 1:2:3 G-quadruplex, as the signals for the imino protons of G12 and G13 were either very weak or missing (Figure 1C), Pu22-T15T16 can form a single G-quadruplex in K^+^ (Supplementary Figure S4B). The 1D ^1^H spectrum of the wild-type sequence appears to show a minor species, likely to be the 1:2:3 loop isomer (Supplementary Figure S4B). It is possible that the HMBC experiment of the 6% ^15^N-G-labeled DNA is not sensitive enough to detect the low population of the 1:2:3 loop isomer. The melting temperature of the 1:4:1 G-quadruplex formed in Pu22-T12T13 is 77.3°C, whereas the melting temperature of the 1:2:3 G-quadruplex is 73°C (Table 1). The melting temperature of the wild-type VEGF-Pu22 is 77.9°C (Table 1), which is close to that of the major conformation 1:4:1 G-quadruplex. The 4°C difference in T_m_ may explain the major formation of the 1:4:1 G-quadruplex in the VEGF promoter sequence.

**Table 1.** Melting temperature (*T*_m_) values for various VEGF 22-mer DNA sequences^a^

DNA	Loop isomer	T_m_ (°C)
VEGF-Pu22	1:4:1	77.9
Pu22-T13	1:4:1	77.1
Pu22-T12T13	1:4:1	77.3
Pu22-T15	1:2:3	73.4
Pu22-T15T16	1:2:3	73

^a^10 mM Tris buffer (pH 7.2), 50 mM potassium chloride, heating rate at 2°C/min.

### Complete NMR spectra assignment of the major VEGF promoter G-quadruplex

**Figure 2.**
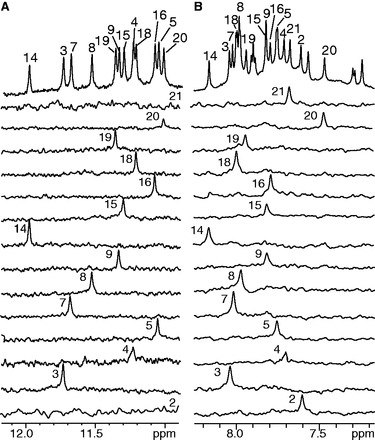
Imino (**A**) and aromatic (**B**) proton assignments of Pu22-T12T13 using 1D ^15^N-edited HMQC experiments on site-specific labeled oligonucleotides. Conditions: 25 mM K-phosphate, 70 mM KCl (pH 7.0), 25°C.

**Figure 3.**
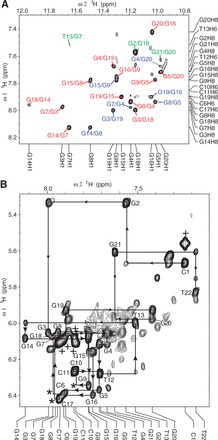
(**A**) The H8-H1 region of the 2D-NOESY spectrum of Pu22-T12T13 in H_2_O at 25°C. Intra-tetrad NOEs are in red, inter- tetrad NOEs are in blue, and NOEs with flanking bases are in green. (**B**) The H1′-H8 region with sequential assignment pathway. Missing connectivities are labeled with asterisks. The cytosine H5-H6 NOEs are labeled with ‘+’.

NMR experiments of Pu22-T12T13 were carried out in 95 mM K^+^ solution. We have also examined this sequence in the physiologically relevant 140 mM K^+^ concentration, which gave rise to the same NMR spectrum (Supplementary Figure S5). The guanine imino and H8 protons of Pu22-T12T13 were assigned using ^15^N-edited HMQC (Figure 2) (36,37). The absence of imino protons for G2 and G21 (Figure 2A) indicated that G2 and G21 were not involved in the G-tetrad formation. Noteworthily, the imino protons of G14, G15 and G16 of Pu22-T12T13 (Figure 1B) are almost at the same locations as those of the wild-type VEGF-Pu22 (Figure 1C). The G-quadruplex formed in Pu22-T12T13 appears to be of monomeric nature as shown by the NMR stoichiometry titration experiment at the melting temperature (Supplementary Figure S6). Pu22-T12T13 forms a parallel-stranded intramolecular G-quadruplex with 1:4:1 loop-size arrangement (Figure 1D). This folding topology was determined by NOE connectivities of guanine imino and H8 protons. In a G-tetrad plane with a Hoogsteen H-bond network, the NH1 of a guanine is in close spatial vicinity to the NH1s of the adjacent guanines and to the H8 of one of the adjacent guanines (Figure 1E). For example, the NOEs of G18H8/G14H1, G14H8/G7H1, G7H8/G3H1 and G3H8/G18H1 (Figure 3A) defined the tetrad plane of G3-G7-G14-G18. The other two tetrad-planes, G4-G8-G15-G19 and G5-G9-G16-G20, were determined in a similar way.

**Figure 4.**
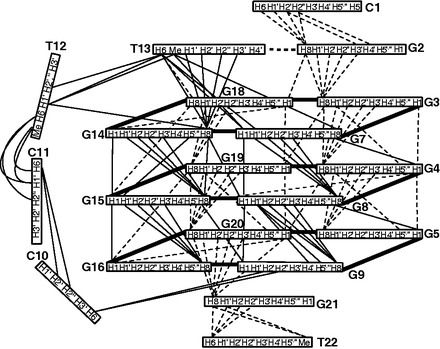
Schematic diagram of inter-residue NOE connectivities of Pu22-T12T13.

Complete proton assignment of Pu22-T12T13 was accomplished by sequential assignment (Figure 3) using 2D COSY, TOCSY and NOESY at different temperatures in both H_2_O and D_2_O (35–37). The chemical shifts of all proton resonances are listed in Table 2. All of the residues appear to adopt anti conformation based on the intensities of intra-residue H8-H1’ cross peaks (Figure 3B). Critical inter-residue NOE interactions are summarized in Figure 4 and define the overall structure of the VEGF promoter G-quadruplex.

**Table 2.** Proton chemical shifts for Pu22-T12T13 at 25°C^a^

Residue	H6/H8	Me/H5/H1	H1′	H2′/H2′′	H3′	H4′	H5′/H5′′
C1	7.24	5.55	5.67	1.17/1.95	4.3	4.23	3.37/3.77
G2	7.57	10.65^b^	5.33	2.48/2.51	4.75	4.44	3.78/4.07
G3	8.00	11.69	6.06	2.76/2.98	4.97	4.40	4.03/4.24
G4	7.67	11.19	6.11	2.58/2.88	4.96	4.49	4.23/4.26
G5	7.72	11.01	6.36	2.63/2.49	5.03	4.56	4.36/4.22
C6	7.91	6.09	6.39	2.33/2.65	4.96	4.51	4.22/4.28
G7	7.97	11.64	6.08	2.41/2.87	5.10	4.40	4.18/4.28
G8	7.94	11.49	6.05	2.64/2.80	5.00	4.47	4.24/4.31
G9	7.78	11.29	6.34	2.54/2.52	4.91	4.41	4.15/4.27
C10	7.85	6.10	6.26	2.25/2.53	4.77	4.41	3.99/4.13
C11	7.86	6.08	6.27	2.30/2.56	4.81	4.33	4.05/4.11
T12	7.71	1.87	6.28	2.36/2.51	4.85	4.32	4.10/4.24
T13	7.51	1.57	5.99	2.21/2.39	4.86	4.20	3.93/4.01
G14	8.13	11.94	6.08	2.77/3.01	4.96	4.39	4.02/4.27
G15	7.77	11.26	6.16	2.67/2.89	4.95	4.51	4.16/4.28
G16	7.75	11.03	6.39	2.60/2.53	5.03	4.58	4.22/4.34
C17	7.94	6.11	6.42	2.33/2.67	5.03	4.56	4.22/4.30
G18	7.96	11.16	6.07	2.35/2.81	5.09	4.40	4.19/4.28
G19	7.9	11.32	5.93	2.62	5.00	4.45	4.13/4.19
G20	7.42	10.97	5.97	2.34/2.72	4.87	4.42	4.13/4.20
G21	7.63	10.20^b^	5.6	2.26/2.36	4.73	4.43	4.12/4.19
T22	7.18	1.40	5.83	1.97/2.02	4.31	3.92	3.66/3.83

^a^The chemical shifts are measured in 25 mM K- phosphate, 70 mM KCl (pH 7.0) referenced to DSS.

^b^Chemical shift measured at 2°C.

Complete spectral assignment (Supplementary Figure S7) was also accomplished for Pu22-T12T13A2A21 with additional G-to-A mutations at G2 and G21.

### NOE interactions define the overall structure of the VEGF G-quadruplex and show specific interactions between the 4-nt middle loop and flanking sequences

The guanines on each of the four G-strands are well stacked, as indicated by the clear NOE connections of adjacent guanine H8 protons, such as G3H8/G4H8, G14H8/G15H8 and G19H8/G20H8 (Figure 4). The sequential NOE connectivities along each G-strand are clearly observed for (n)GH8 and (n-1)GH1′/H2′/H2″/H3′, typical for right-handed DNA backbone conformation (Figures 3B and 4). Inter-tetrad NOE connectivities of non-sequential guanines of G-strands, such as G3H8/G19H1 and G4H8/G20H1, G7H8/G4H1 and G8H8/G5H1, G14H8/G8H1 and G15H8/G9H1, and G19H8/G16H1, were clearly observed (Figure 3A), supporting both the folding structure and the right-handed twist of the G-strands. The clear NOE cross-peaks between sugar H1′ and (n+1) H4′ or H5′,′′ e.g., G3H1′/G4H5′,′′, G4H1′/G5H5′,′′, G8H1′/G9H4′, H5′,′′, G15H1′/G16H5′,′′ and G19H1′/G20H5′,′′, indicated that the sugar backbones of the G-strands are more compact than regular B-DNA (35,37).

The sequential NOE cross-peaks are absent or weak at the three double-chain-reversal loops, i.e. C6, C10-C11-T12-T13 and C17 (Figure 3B). The two 1-nt loop cytosines (C6 and C17) show similar chemical shifts, which are both downfield-shifted due to the groove location. Unexpectedly, T13 of the 4-nt loop appears to stack well with the 5′-tetrad: in addition to sequential NOEs at the T13-G14 step, such as G14H8/T13H6, G14H8/T13H1′, G14H8/T13H2′,′′ and G14H8/T13H3′ (Figure 4), a clear NOE is observed between T13H6/G7H1 (Figure 3A), indicating that T13 stacks well with G14 towards the G7 side. Sequential NOEs for stacking interactions are not observed for the other three residues of the 4-nt loop, and clearly downfield-shifted chemical shifts are suggestive of their groove location. A number of NOEs are observed for the two flanking sequences, both of which appear to adopt well-stacking conformations. For the 5′-flanking C1-G2 segment, sequential NOEs are observed at the G2-G3 step, such as G3H8/G2H8, G3H8/G2H1′, H2′,′′ and H3′, as well as at the C1-G2 step (Figures 3B and 4). Surprisingly, the NOE between G2H8/G18H1 was strong, indicating that G2 stacks completely above the 5′-tetrad with its H8 end positioned right above G18H1. Similar sequential NOEs are observed for the 3′-flanking G21-T22 segment, i.e. at G21-G20 and T22-G21 steps (Figures 3B and 4). A clear NOE observed between G21H8/G20H1 (Figure 3A) indicates that G21 stacks well with G20.

### NOE-refined solution structure of the VEGF G-quadruplex shows unique capping structure involving both the 4-nt middle loop and the two flanking segments

**Figure 5.**
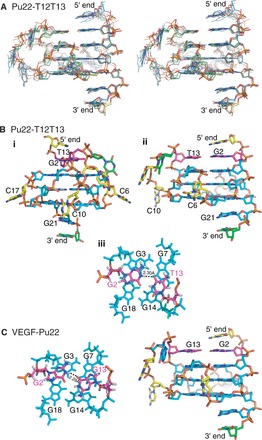
(**A**) Stereo view of 10 lowest energy structures of the Pu22-T12T13 G-quadruplex by NOE-restrained structure calculation. (**B**) A representative structure of the NMR-refined Pu22-T12T13 G-quadruplex in two different views (i, ii); and the 5′-end view of the capping structure (magenta) that involves the 4-nt middle loop and 5′-flanking segment (iii). (**C**) The molecular model of the wild-type VEGF-Pu22 G-quadruplex by unrestrained molecular dynamics simulation (right). The 5′-end views of the capping structure (magenta) is also shown (left).

Solution structures of the Pu22-T12T13 G-quadruplex were calculated using a NOE-restrained distance geometry (DGSA) and restrained molecular dynamics (RMD) approach (Figure 5, PDB ID 2m27), starting from an arbitrary extended single-stranded DNA. A total of 407 NOE distance restraints, including 145 inter-residue NOE interactions, were used in the NOE-restrained structure calculation (Supplementary Table S1). Dihedral restraints are used for the anti glycosidic torsion angle (χ) for loop residues. The stereo view of the 10 lowest energy structures is shown in Figure 5A. The structure statistics are listed in Supplementary Table S1. Pu22-T12T13 forms a well-defined parallel-stranded G-quadruplex structure with three tetrads. The two 1-nt loops are located in the groove and adopt similar conformations, with extended sugar backbone and the cytosine base sticking out to the solvent (Figure 5B). The 4-nt double-chain-reversal loop, C10-C11-T12-T13, interestingly, adopts a unique conformation (Figure 5B). The T13 base stacks over the G14 base and appears to be hydrogen-bonded with the G2 base of the 5′ flanking segment (Figure 5B-iii). The hydrogen-bond interaction was supported by NMR, i.e., the G2 imino proton was detected at 2°C at ∼10.8 ppm (Supplementary Figure S2). The G2:T13 base pair appears to completely stack over the 5′ G-tetrad (Figure 5B-iii) and thus would experience strong ring-current effect. This is shown by the NMR data, i.e. a clear upfield-shifting of the chemical shifts for sugar protons of G2 and T13, e.g. G2H1′, (Figure 3B, Table 2). The other three residues, C10, C11 and T12, are located in the groove to connect the now four-layer structure (three G-tetrads plus one G: T base pair) with the C9 and C10 bases pointing out to the solvent. The T13, which is involved in the G2:T13 base pair capping structure, is a mutation from the wild-type G13. To examine the G-quadruplex formed in the wild-type sequence VEGF-Pu22, we took the G-quadruplex structure formed in Pu22-T12T13 and replaced T12 and T13 with the wild-type G12 and G13 residues. We carried out energy minimization followed by unrestrained molecular dynamics simulation for 25 ps at 300 K. Notably, a hydrogen-bonded G2:G13 base pair can be nicely formed in the wild-type sequence to cap the VEGF G-quadruplex quadruplex (Figure 5C). We have collected 2D NOESY data with a 50 ms mixing time for the wild-type sequence VEGF-Pu22. Similar to what was observed in the Pu22-T12T13 sequence, no *syn* conformation was observed for any nucleotide in the VEGF-Pu22 sequence (Supplementary Figure S8).

## DISCUSSION

The NMR results in the present study unequivocally demonstrated that the major intramolecular G-quadruplex formed in the VEGF proximal promoter in K^+^ solution is a parallel-stranded structure with a 1:4:1 loop-size arrangement. The minor species, a 1:2:3 loop isomer (Supplementary Figure S4), could not be detected in the wild-type sequence VEGF-Pu22 by NMR, as the imino proton of G13, which is required for the core-tetrad of the 1:2:3 loop isomer (Supplementary Figure S4), was not detected, even at 2°C (Figure 1C and Supplementary Figure S2). The T_m_ of the 1:2:3 loop isomer was shown to be 4°C lower than that of the 1:4:1 loop isomer, which may explain the major formation of the 1:4:1 G-quadruplex in the VEGF promoter sequence.

**Figure 6.**
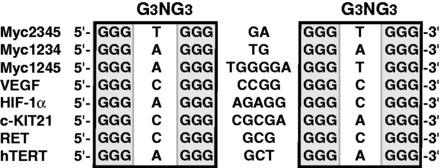
Parallel-stranded G-quadruplex-forming promoter sequences.

Parallel-stranded structures have been found to be common in the human promoter G-quadruplexes, such as c-MYC (35,44,45), HIF-1α (46), c-KIT21 (47), RET (48) and hTERT (49,50). Importantly, all of these parallel-stranded promoter G-quadruplexes contain three tetrads and two 1-nt loops (first and third), but a variable-length middle loop (Figure 6) (26,32). We have previously determined the molecular structure of the major G-quadruplex formed in the c-MYC promoter, a three-tetrad parallel structure with 1:2:1 loop-size arrangement (35), which shows that the 1-nt loop is highly favored in parallel-stranded G-quadruplexes because of the right-handed twist of the adjacent G-strands. Although the VEGF G-quadruplex also contains the 1-nt first and third loops, the middle loop of the VEGF G-quadruplex is 4 nt long. Significantly, unlike the 2-nt middle loop of the MYC G-quadruplex that stays in the groove, the 4-nt middle loop of the VEGF G-quadruplex stretches over the 5′ tetrad to form a unique capping structure with the flanking segment. This capping structure was observed in the Pu22-T12T13 sequence with two G-to-T mutations at the 12 and 13 positions; a similar capping structure was also shown to form in the wild-type sequence VEGF-Pu22 using unrestrained molecular dynamics simulation. It is noted that, although the two capping structures in the wild-type and mutant sequences are similar, there appear to be differences in their respective conformations. For example, the G13:G2 capping structure is larger than that of the T13:G2 capping structure formed in the mutant sequence and would thus cover more of the top G-tetrad. In addition, the groove-located wild-type G12 residue also likely to possess a stronger ring-current effect on G7 than that of the mutated T12 (Figure 5), which could explain the observed upfield-shifting of the resonance of G7 imino proton in VEGF-Pu22 as compared with Pu22-T12T13 (Figure 1 and Supplementary Figure S4). As such, the 4-nt middle loop of the VEGF G-quadruplex appears to play a critical role in forming the specific capping structure and stabilizing the most favored folding structure. This capping structure represents a unique, VEGF sequence-specific loop interaction and distinguishes the VEGF G-quadruplex from other parallel-stranded structures, such as the MYC G-quadruplex whose capping structures are formed solely by the flanking segments due to the short 2-nt middle loop (35). The specific capping structure of the VEGF promoter G-quadruplex may be recognized by small molecule or protein ligands, and the molecular structure described in this study could provide a starting point for structure-based rational design of quadruplex-interactive small molecules targeting VEGF. In conclusion, although parallel structures are common to the promoter G-quadruplexes, our study indicates that each G-quadruplex is likely to adopt unique capping structures by its specific variable middle loop and flanking segments, which together determine the overall structure and specific interactions with small molecules or proteins.

## ACCESSION NUMBERS

PDB ID 2m27

## SUPPLEMENTARY DATA

Supplementary Data are available at NAR Online.

## FUNDING

National Institutes of Health (NIH) [CA122952 and GM083117]. Funding for open access charge: (NIH) [GM083117].

*Conflict of interest statement*. None declared.

## Supplementary Material

Supplementary Data

## References

[1] Weidner N, Folkman J, Pozza F, Bevilacqua P, Allred EN, Moore DH, Meli S, Gasparini G (1992). Tumor angiogenesis - a new significant and independent prognostic indicator in early-stage breast-carcinoma. J. Natl Cancer Inst..

[2] Ferrara N (1996). Vascular endothelial growth factor. Eur. J. Cancer.

[3] Folkman J (2002). Role of angiogenesis in tumor growth and metastasis. Semin. Oncol..

[4] Jain RK (2002). Tumor angiogenesis and accessibility: role of vascular endothelial growth factor. Semin. Oncol..

[5] Schafer G, Cramer T, Suske G, Kemmner W, Wiedenmann B, Hocker M (2003). Oxidative stress regulates vascular endothelial growth factor-a gene transcription through Sp1-and Sp3-dependent activation of two proximal GC-rich promoter elements. J. Biol. Chem..

[6] Maeno T, Tanaka T, Sando Y, Suga T, Maeno Y, Nakagawa J, Hosono T, Sato M, Akiyama H, Kishi S (2002). Stimulation of vascular endothelial growth factor gene transcription by all trans retinoic acid through Sp1 and Sp3 sites in human bronchioloalveolar carcinoma cells. Am. J. Respir. Cell Mol. Biol..

[7] Chen HZ, Ye DF, Xie X, Chen BY, Lu WG (2004). VEGF, VEGFRs expressions and activated STATs in ovarian epithelial carcinoma. Gynecol. Oncol..

[8] Shi Q, Le XD, Abbruzzese JL, Peng ZH, Qian CN, Tang HM, Xiong QH, Wang BL, Li XC, Xie KP (2001). Constitutive Sp1 activity is essential for differential constitutive expression of vascular endothelial growth factor in human pancreatic adenocarcinoma. Cancer Res..

[9] Pal S, Datta K, Mukhopadhyay D (2001). Central role of p53 on regulation of vascular permeability factor/vascular endothelial growth factor (VPF/VEGF) expression in mammary carcinoma. Cancer Res..

[10] Grugel S, Finkenzeller G, Weindel K, Barleon B, Marme D (1995). Both V-Ha-Ras and V-Raf stimulate expression of the vascular endothelial growth-factor in Nih 3t3 Cells. J. Biol. Chem..

[11] Forsythe JA, Jiang BH, Iyer NV, Agani F, Leung SW, Koos RD, Semenza GL (1996). Activation of vascular endothelial growth factor gene transcription by hypoxia-inducible factor 1. Mol.Cell Biol..

[12] Cohen T, Nahari D, Cerem LW, Neufeld G, Levi BZ (1996). Interleukin 6 induces the expression of vascular endothelial growth factor. J. Biol. Chem..

[13] Finkenzeller G, Sparacio A, Technau A, Marme D, Siemeister G (1997). Sp1 recognition sites in the proximal promoter of the human vascular endothelial growth factor gene are essential for platelet-derived growth factor-induced gene expression. Oncogene.

[14] Martinybaron G, Marme D (1995). Vegf-mediated tumor angiogenesis - a new target for cancer-therapy. Curr. Opin. Biotechnol..

[15] Ellis LM, Hicklin DJ (2008). VEGF-targeted therapy: mechanisms of anti-tumour activity. Nat. Rev. Cancer.

[16] Eichholz A, Merchant S, Gaya AM (2010). Anti-angiogenesis therapies: their potential in cancer management. OncoTargets Ther..

[17] Bikfalvi A, Bicknell R (2002). Recent advances in angiogenesis, anti-angiogenesis and vascular targeting. Trends Pharmacol. Sci..

[18] Sepp-Lorenzino L, Thomas KA (2002). Antiangiogenic agents targeting vascular endothelial growth factor and its receptors in clinical development. Expert Opin. Investig. Drugs.

[19] Niederman TMJ, Ghogawala Z, Carter BS, Tompkins HS, Russell MM, Mulligan RC (2002). Antitumor activity of cytotoxic T lymphocytes engineered to target vascular endothelial growth factor receptors. Proc. Natl Acad. Sci. USA.

[20] Jin N, Chen W, Blazar BR, Ramakrishnan S, Vallera DA (2002). Gene therapy of murine solid tumors with T cells transduced with a retroviral vascular endothelial growth factor-immunotoxin target gene. Hum. Gene Ther..

[21] Goodman VL, Rock EP, Dagher R, Ramchandani RP, Abraham S, Gobburu JVS, Booth BP, Verbois SL, Morse DE, Liang CY (2007). Approval summary: sunitinib for the treatment of imatinib refractory or intolerant gastrointestinal stromal tumors and advanced renal cell carcinoma. Clin. Cancer Res..

[22] Kane RC, Farrell AT, Saber H, Tang S, Williams G, Jee JM, Liang C, Booth B, Chidambaram N, Morse D (2006). Sorafenib for the treatment of advanced renal cell carcinoma. Clin. Cancer Res..

[23] Ferrara N, Hillan KJ, Gerber HP, Novotny W (2004). Discovery and development of bevacizumab, an anti-VEGF antibody for treating cancer. Nat. Rev. Drug Discov..

[24] Balasubramanian S, Hurley LH, Neidle S (2011). Targeting G-quadruplexes in gene promoters: a novel anticancer strategy? *Nat*. Rev. Drug Discov..

[25] Brooks TA, Hurley LH (2010). Targeting MYC expression through G-quadruplexes. Genes Cancer.

[26] Yang DZ, Okamoto K (2010). Structural insights into G-quadruplexes: towards new anticancer drugs. Future Med. Chem..

[27] Biffi G, Tannahill D, McCafferty J, Balasubramanian S (2013). Quantitative visualization of DNA G-quadruplex structures in human cells. Nat. Chem..

[28] Sun DY, Guo KX, Rusche JJ, Hurley LH (2005). Facilitation of a structural transition in the polypurine/polypyrimidine tract within the proximal promoter region of the human VEGF gene by the presence of potassium and G-quadruplex-interactive agents. Nucleic Acids Res..

[29] Guo K, Gokhale V, Hurley LH, Sun D (2008). Intramolecularly folded G-quadruplex and i-motif structures in the proximal promoter of the vascular endothelial growth factor gene. Nucleic Acids Res..

[30] Sun D, Guo K, Shin YJ (2011). Evidence of the formation of G-quadruplex structures in the promoter region of the human vascular endothelial growth factor gene. Nucleic Acids Res..

[31] Sun D, Liu WJ, Guo KX, Rusche JJ, Ebbinghaus S, Gokhale V, Hurley LH (2008). The proximal promoter region of the human vascular endothelial growth factor gene has a G-quadruplex structure that can be targeted by G-quadruplex-interactive agents. Mol.Cancer Ther..

[32] Chen Y, Yang DZ (2012). Sequence, stability, and structure of G-quadruplexes and their interactions with drugs. Curr. Protoc. Nucleic Acid Chem..

[33] Dai J, Dexheimer TS, Chen D, Carver M, Ambrus A, Jones RA, Yang DZ (2006). An intramolecular G-quadruplex structure with mixed parallel/antiparallel G-strands formed in the human BCL-2 promoter region in solution. J. Am. Chem. Soc..

[34] Dai J, Carver M, Punchihewa C, Jones RA, Yang DZ (2007). Structure of the Hybrid-2 type intramolecular human telomeric G-quadruplex in K+ solution: insights into structure polymorphism of the human telomeric sequence. Nucleic Acids Res..

[35] Ambrus A, Chen D, Dai J, Jones RA, Yang DZ (2005). Solution structure of the biologically relevant G-quadruplex element in the human c-MYC promoter. Implications for G-quadruplex stabilization. Biochemistry.

[36] Zhang Z, Dai J, Veliath E, Jones RA, Yang DZ (2010). Structure of a two-G-tetrad intramolecular G-quadruplex formed by a variant human telomeric sequence in K+ solution: insights into the interconversion of human telomeric G-quadruplex structures. Nucleic Acids Res..

[37] Mathad RI, Hatzakis E, Dai J, Yang DZ (2011). c-MYC promoter G-quadruplex formed at the 5′-end of NHE III1 element: insights into biological relevance and parallel-stranded G-quadruplex stability. Nucleic Acids Res..

[38] Hatzakis E, Okamoto K, Yang DZ (2010). Thermodynamic stability and folding kinetics of the major g-quadruplex and its loop isomers formed in the nuclease hypersensitive element in the human c-Myc promoter: effect of loops and flanking segments on the stability of parallel-stranded intramolecular G-quadruplexes. Biochemistry.

[39] Ambrus A, Chen D, Dai J, Bialis T, Jones RA, Yang DZ (2006). Human telomeric sequence forms a hybrid-type intramolecular G-quadruplex structure with mixed parallel/antiparallel strands in potassium solution. Nucleic Acids Res..

[40] Szewczak AA, Kellogg GW, Moore PB (1993). Assignment of NH resonances in nucleic acids using natural abundance 15N-1H correlation spectroscopy with spin-echo and gradient pulses. FEBS Lett..

[41] Zhao H, Pagano AR, Wang WM, Shallop A, Gaffney BL, Jones RA (1997). Use of a C-13 atom to differentiate two N-15-labeled nucleosides. Syntheses of [(NH2)-N-15]-adenosine, [1,NH2-N-15(2)]- and [2-C-13-1,NH2-N-15(2)]-guanosine, and [1,7,NH2-N-15(3)]- and [2-C-13-1,7,NH2-N-15(3)]-2'-deoxyguanosine. J. Org. Chem..

[42] Sklenar V, Bax A (1987). Spin-echo water suppression for the generation of pure-phase two-dimensional NMR spectra. J. Magn. Reson..

[43] Brünger AT (1993). X-PLOR Version 3.1: A System for X-ray Crystallography and NMR.

[44] Seenisamy J, Rezler EM, Powell TJ, Tye D, Gokhale V, Joshi CS, Siddiqui-Jain A, Hurley LH (2004). The dynamic character of the G-quadruplex element in the c-MYC promoter and modification by TMPyP4. J. Am. Chem. Soc..

[45] Phan AT, Modi YS, Patel DJ (2004). Propeller-type parallel-stranded G-quadruplexes in the human c-myc promoter. J. Am. Chem. Soc..

[46] De Armond R, Wood S, Sun DY, Hurley LH, Ebbinghaus SW (2005). Evidence for the presence of a guanine quadruplex forming region within a polypurine tract of the hypoxia inducible factor 1 alpha promoter. Biochemistry.

[47] Fernando H, Reszka AP, Huppert J, Ladame S, Rankin S, Venkitaraman AR, Neidle S, Balasubramanian S (2006). A conserved quadruplex motif located in a transcription activation site of the human c-kit oncogene. Biochemistry.

[48] Guo K, Pourpak A, Beetz-Rogers K, Gokhale V, Sun D, Hurley LH (2007). Formation of pseudosymmetrical G-quadruplex and i-motif structures in the proximal promoter region of the RET oncogene. J. Am. Chem. Soc..

[49] Palumbo SL, Ebbinghaus SW, Hurley LH (2009). Formation of a unique end-to-end stacked pair of G-Quadruplexes in the hTERT core promoter with implications for inhibition of telomerase by G-Quadruplex-interactive ligands. J. Am. Chem. Soc..

[50] Lim KW, Lacroix L, Yue DJE, Lim JKC, Lim JMW, Phan AT (2010). Coexistence of two distinct G-Quadruplex conformations in the hTERT promoter. J. Am. Chem. Soc..

