# Supplementary material for: Solution structure of the major G-quadruplex formed in the human VEGF promoter in K+: insights into loop interactions of the parallel G-quadruplexes
Source: Nucleic Acids Res. 2013 Sep 4;41(22):10584–92. doi: 10.1093/nar/gkt784 (PMC3905851; doi:10.1093/nar/gkt784)
Supplement: Supplementary Data [file supp_41_22_10584__index.html]

Solution structure of the major G-quadruplex formed in the human VEGF promoter in K+: insights into loop interactions of the parallel G-quadruplexes — Solution structure of the major G-quadruplex formed in the human VEGF promoter in K+: insights into loop interactions of the parallel G-quadruplexes — Supplementary Data 

# Solution structure of the major G-quadruplex formed in the human VEGF promoter in K+: insights into loop interactions of the parallel G-quadruplexes

## Supplementary Data

files

**Files in this Data Supplement:**

- Supplementary Data - pdf file
