# Supplementary material for: Solution structure of the major G-quadruplex formed in the human VEGF promoter in K+: insights into loop interactions of the parallel G-quadruplexes
Source: Nucleic Acids Res. 2013 Sep 4;41(22):10584–92. doi: 10.1093/nar/gkt784 (PMC3905851; doi:10.1093/nar/gkt784)
Supplement: Supplementary Data [file supp_gkt784_nar-01291-f-2013-File008.pdf]

Solution structure of the major G-quadruplex formed in the human VEGF promoter in K^+: Insights into loop interactions of the parallel G-quadruplexes

Prashansa Agrawal [‡], Emmanuel Hatzakis [‡], Kexiao Guo [‡], Megan Carver [‡], Danzhou Yang[‡§‖†*]

Supplementary Information

RUNNING TITLE: human VEGF Promoter G-quadruplex Structure

Table S1. Structural statistics for Pu22-T12T13 [a]

Structural statistics	
NMR distance constraints	
Distance restraints	407
Intraresidue	262
Interresidue	145
Sequential (|i – j| = 1)	115
Non-sequential (|i – j| > 1)	30
Hydrogen bonds	36
Deviations from standard geometry	
Bond length (Å)	0.0071 ± 0.0001
Bond angle (°)	1.29 ± 0.01
NOE violations	
numbers (>0.2Å)	0.025 ± 0.005
pairwise r.m.s.d. of heavy atoms (Å)	
G-tetrads	0.41±0.20
With C6	0.46±0.21
With C17	0.59±0.22
With C10/C11/T12/T13	0.97±0.61
All	1.10±0.51

[a] The ensemble of 10 structures is selected based on both the minimal energy terms and number of NOE violations.

FIGURE LEGENDS

FIGURE S1. CD spectra of VEGF-Pu22 and Pu22-T12T13 in the presence of 10 mM K-phosphate buffer, 40 mM potassium chloride (pH 7.2), 25 °C.

FIGURE S2. Imino proton resonance assignments of G2, G21, and G13 in the wild-type VEGF-Pu22 sequence using 1D ^{15}N-edited experiments on site-specific labeled DNA at 2 °C. The top spectrum is the imino region of the regular 1D proton spectrum; other spectra are 1D HMQC experiments recorded on site-specific labeled DNA. Experimental conditions: 25 mM K-phosphate, 70 mM KCl, pH 7.0, 0.2 mM DNA.

FIGURE S3. The wild-type VEGF-Pu22 sequence and its modifications (top), and imino regions of 1D ^{1}H NMR spectra of VEGF-Pu22 sequences. Experimental conditions: 25 mM K-phosphate, 70 mM KCl, pH 7.0, 25 °C.

FIGURE S4. (A) The wild-type VEGF-Pu22 sequence and its modifications. Schematic drawing of the 1:2:3 VEGF G-quadruplex loop isomer. Red boxes represent guanines involved in tetrad formation. (B) Imino regions of 1D ^{1}H NMR spectra of VEGF-Pu22 sequences. Experimental conditions: 25 mM K-phosphate, 70 mM KCl, pH 7.0, 25 °C.

FIGURE S5. Imino regions of 1D ^{1}H NMR spectra of Pu22-T12T13 in 140 mM K^+ (top) and 95 mM K^+ (bottom) at 25 °C.

FIGURE S6. (A) The variable-temperature study of Pu22-T12T13 by NMR. The melting temperature of Pu22-T12T13 is shown to be around 75 °C, with 50% in folded form. (B) The extended region of 1D 1H NMR spectra of Pu22-T12T13 at various concentrations showing the two peaks from the folded and the melted forms at the melting temperature 75 °C, with the one belonging to the melted form labeled with asterisk and the one belonging to the folded forms labeled with cross. The peak intensities of two resolved peaks from melted and folded forms were used for the calculation to determine stoichiometry. (C) Determination of stoichiometry by NMR titration for Pu22-T12T13 in K^+ solution. The slope of the fitted line is 0.99, meaning that the quadruplex structure existing in solution is unimolecular.

FIGURE S7. The expanded H8-H1 region of the exchangeable 2D NOESY spectrum of VEGF Pu22-T12T13A2A21 at 5°C. Cross-peaks are labeled using the [(H8) guanine residue number/(H1) guanine residue number] pairing system. The inter-residue connectivities within a tetrad plane are labeled in red. Blue color labels represent inter-tetrad connectivities. Green color labels show additional cross peaks between the tetrad and loop/flanking residues. (B) The expanded H8/H6–H1' region of 2D NOESY spectrum of VEGF Pu22-T12T13A2A21 at 25°C with assignments. The sequential assignment pathway is shown. Missing connectivities are shown with asterisks. Conditions: 200 ms mixing time; 25 mM K-phosphate, 70 mM KCl, pH 7.0, 2.0 mM DNA.

FIGURE S8. The expanded H8/H6-H1' region of 2D NOESY spectrum of VEGF-Pu22 with 50 ms mixing-time at 25°C. The cytosine H5-H6 NOEs are labeled.

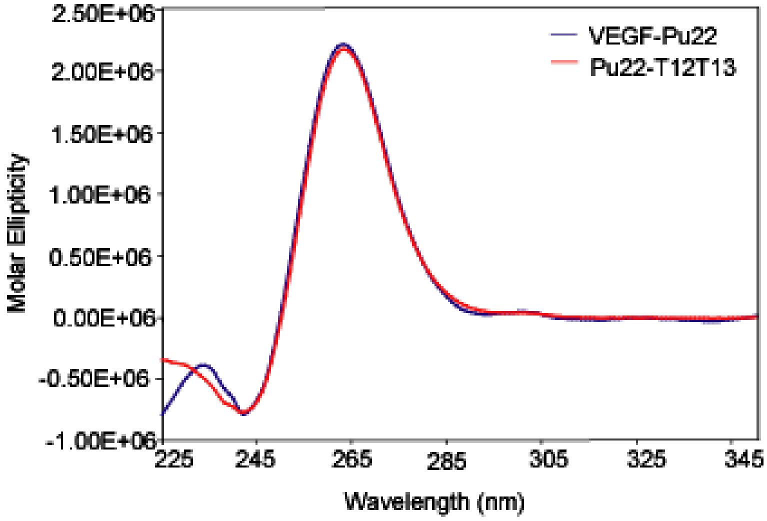
VEGF-Pu22
Pu22-T12T13
2.50E+06
2.00E+06
1.50E+06
1.00E+06
0.50E+06
0.00E+06
-0.50E+06
-1.00E+06
Molar Ellipticity
225
245
265
285
305
325
345
Wavelength (nm)

Figure S1.

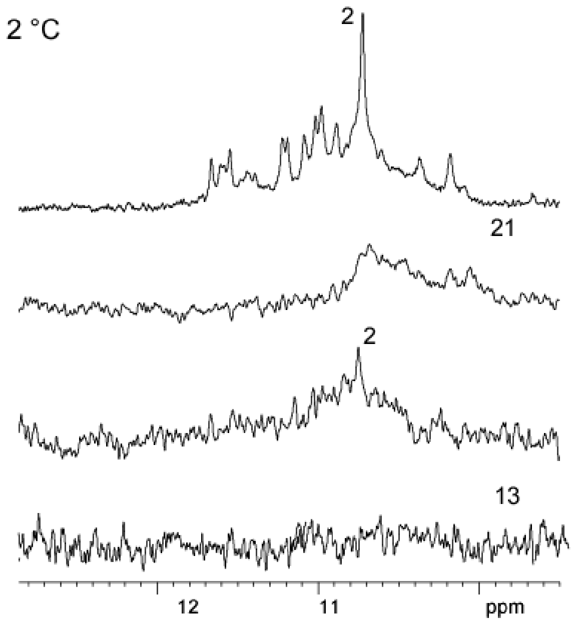

Figure S2.

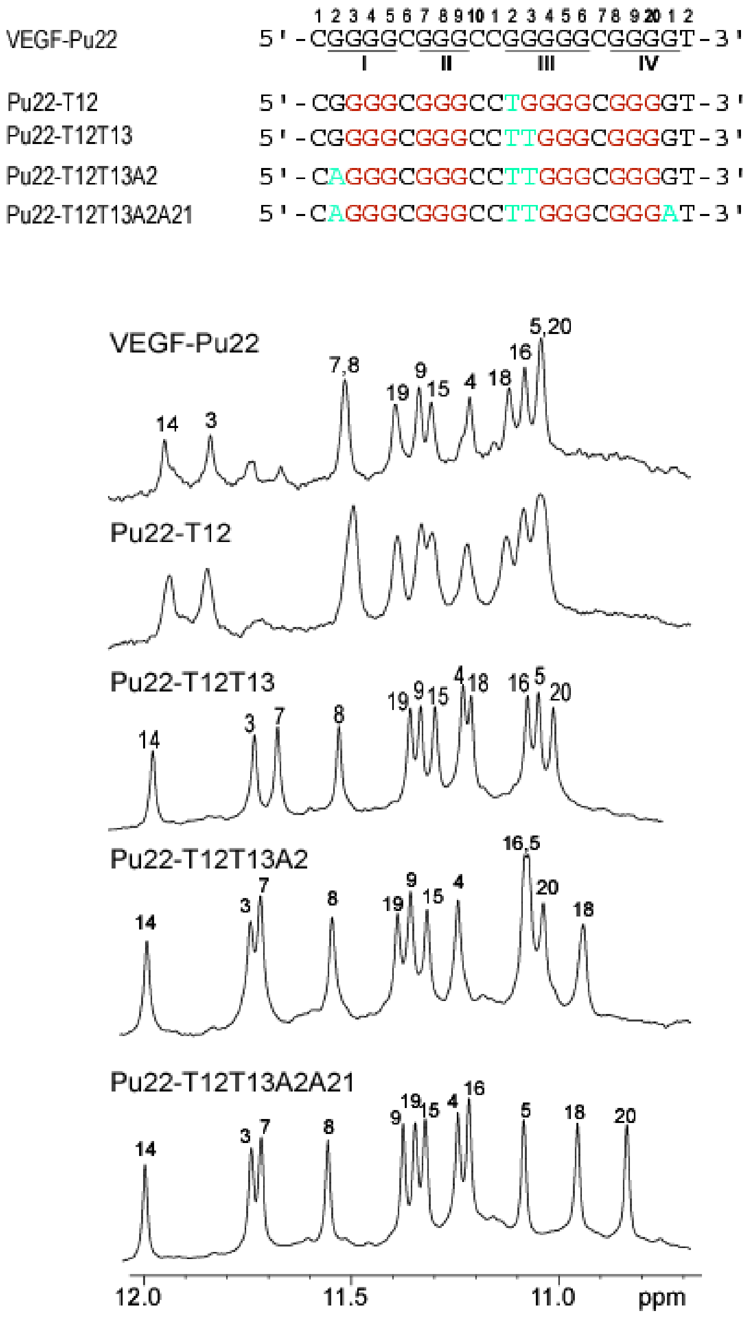
1 2 3 4 5 6 7 8 9 10 1 2 3 4 5 6 7 8 9 20 1 2
VEGF-Pu22 5'-CGGGGCGGGCCGGGGGCGGGGT-3'
I II III IV
Pu22-T12 5'-CGGGGCGGGCCTGGGGCGGGGT-3'
Pu22-T12T13 5'-CGGGGCGGGCCTTGGGCGGGGT-3'
Pu22-T12T13A2 5'-CAGGGCGGGCCTTGGGCGGGGT-3'
Pu22-T12T13A2A21 5'-CAGGGCGGGCCTTGGGCGGGAT-3'
VEGF-Pu22
14 3 7,8 19 9 15 4 18 16 5,20
Pu22-T12
Pu22-T12T13
14 3 7 8 19 9 15 4 18 16 5 20
Pu22-T12T13A2
14 3 7 8 19 9 15 4 16,5 20 18
Pu22-T12T13A2A21
14 3 7 8 9 19 15 4 16 5 18 20
12.0 11.5 11.0 ppm

Figure S3.

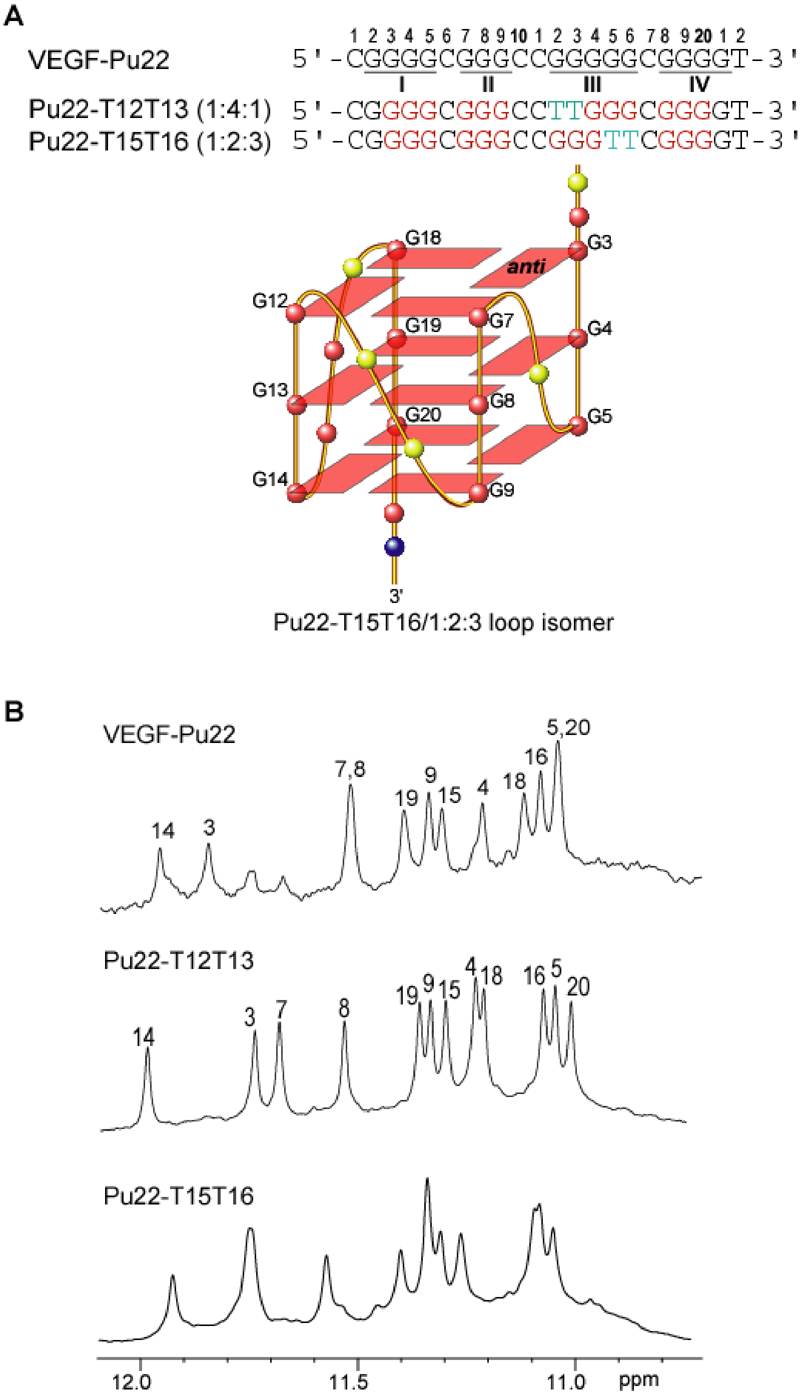
A
1 2 3 4 5 6 7 8 9 10 1 2 3 4 5 6 7 8 9 20 1 2
VEGF-Pu22 5'-CGGGGCGGGCCGGGGGCGGGGT-3'
I II III IV
Pu22-T12T13 (1:4:1) 5'-CGGGGCGGGCCTTGGGCGGGGT-3'
Pu22-T15T16 (1:2:3) 5'-CGGGGCGGGCCGGGTTCGGGGT-3'
G18
G3
anti
G12
G19
G7
G4
G13
G20
G8
G5
G14
G9
3'
Pu22-T15T16/1:2:3 loop isomer
B
VEGF-Pu22
14
3
7,8
19
9
15
4
18
16
5,20
Pu22-T12T13
14
3
7
8
19
9
15
4
18
16
5
20
Pu22-T15T16
12.0
11.5
11.0
ppm

Figure S4.

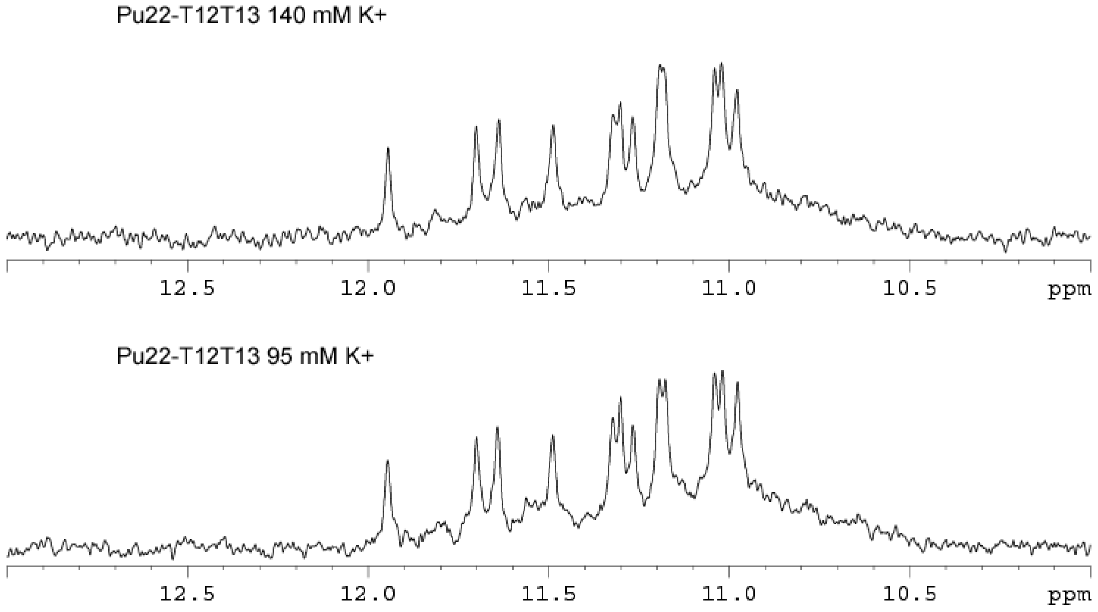
Pu22-T12T13 140 mM K+
12.5
12.0
11.5
11.0
10.5
ppm
Pu22-T12T13 95 mM K+
12.5
12.0
11.5
11.0
10.5
ppm

Figure S5.

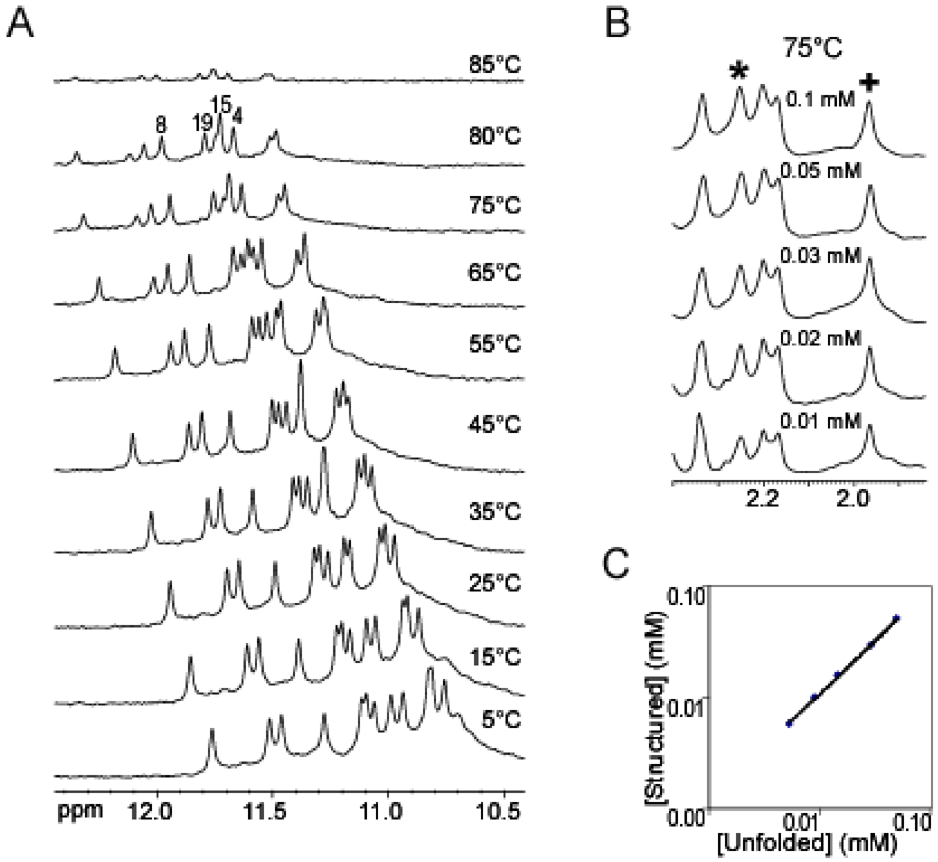
A
85°C
80°C
75°C
65°C
55°C
45°C
35°C
25°C
15°C
5°C
8
19
15
4
ppm
12.0
11.5
11.0
10.5
B
75°C
*
+
0.1 mM
0.05 mM
0.03 mM
0.02 mM
0.01 mM
2.2
2.0
C
0.10
0.01
0.00
0.10
[Structured] (mM)
[Unfolded] (mM)

Figure S6.

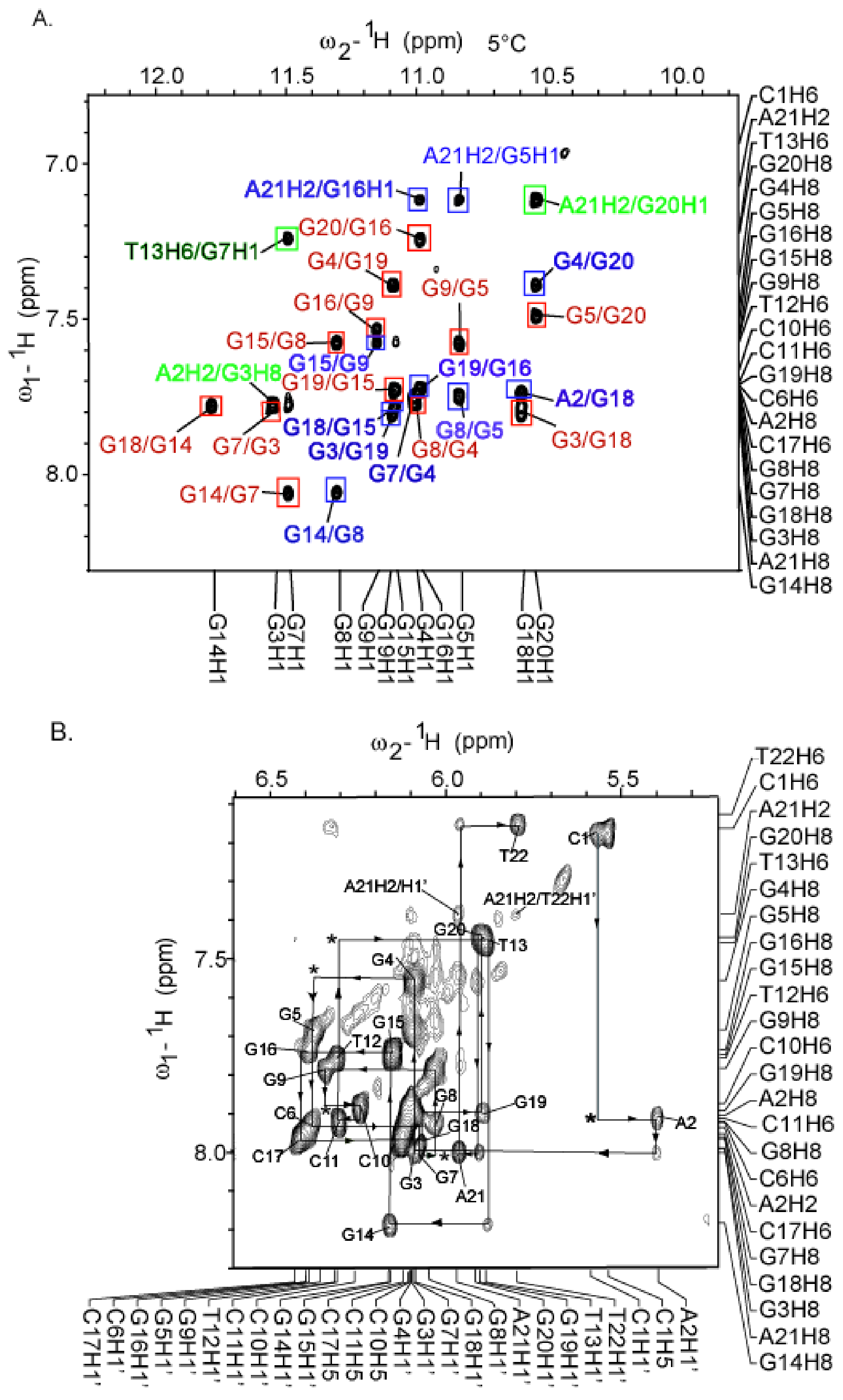
A.
ω2- 1H (ppm) 5°C
ω1- 1H (ppm)
A21H2/G5H1
A21H2/G16H1
A21H2/G20H1
G20/G16
T13H6/G7H1
G4/G19
G4/G20
G9/G5
G16/G9
G5/G20
G15/G8
G15/G9
G19/G16
A2H2/G3H8
G19/G15
A2/G18
G18/G15
G8/G5
G3/G18
G18/G14
G7/G3
G3/G19
G8/G4
G7/G4
G14/G7
G14/G8
B.
ω2- 1H (ppm)
ω1- 1H (ppm)
T22
C1
A21H2/H1'
A21H2/T22H1'
G20
T13
G4
G5
G15
G16
T12
G9
G8
C6
G19
G18
A2
C17
C11
C10
G3
G7
A21
G14

Figure S7.

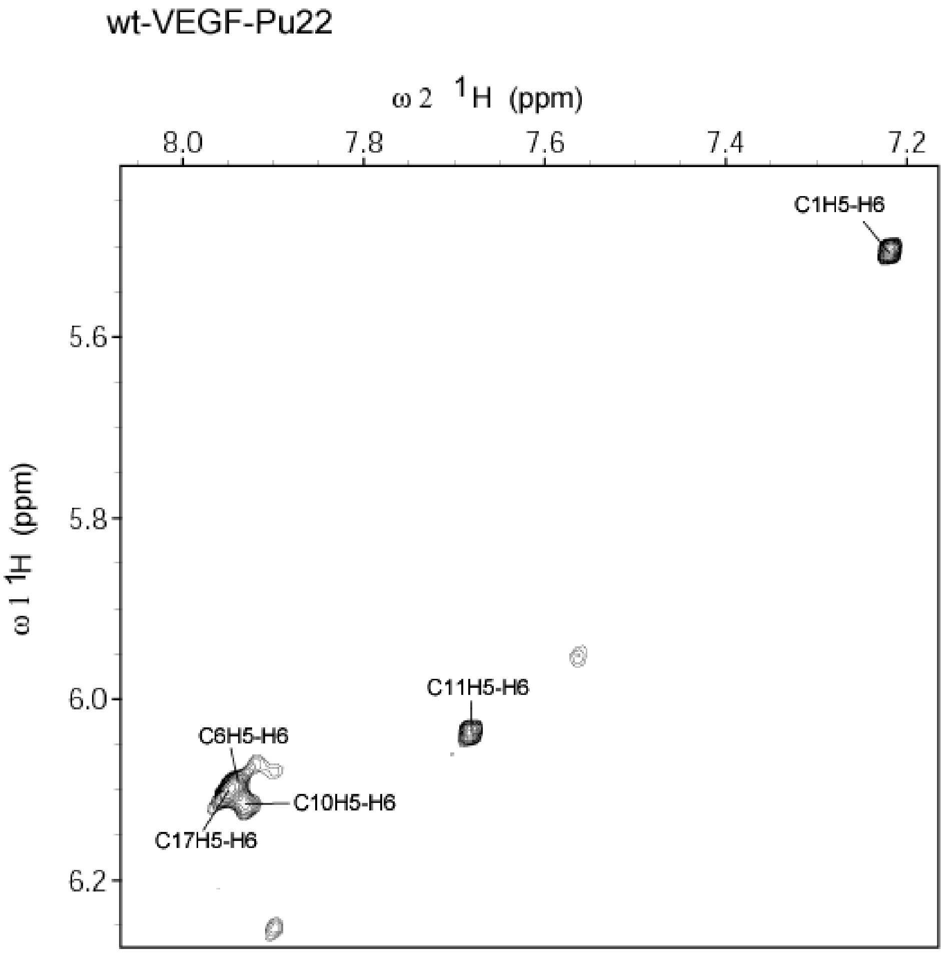
wt-VEGF-Pu22
ω 2 1H (ppm)
8.0
7.8
7.6
7.4
7.2
ω 1 1H (ppm)
5.6
5.8
6.0
6.2
C1H5-H6
C11H5-H6
C6H5-H6
C10H5-H6
C17H5-H6

Figure S8.
